# Association of Hypomagnesemia With Diabetic Complications

**DOI:** 10.7759/cureus.56605

**Published:** 2024-03-20

**Authors:** Syed Khurram Shehzad Kazmi, Mehrin Farooq, Iqra Iftikhar, Naqsh Fatima, Mahwish Shahzad, Asad Ullah Ijaz, Humna Khalid

**Affiliations:** 1 General Medicine, Lahore Medical and Dental College, Lahore, PAK; 2 General Medicine, Ghurki Trust Teaching Hospital, Lahore, PAK; 3 Internal Medicine, Ghurki Trust Teaching Hospital, Lahore, PAK; 4 Internal Medicine, Lahore Medical and Dental College, Lahore, PAK; 5 Medicine, Jinnah Hospital, Lahore, PAK; 6 Biochemistry, Lahore Medical and Dental College, Lahore, PAK; 7 Internal Medicine/Dermatology, Bahawal Victoria Hospital, Bahawalpur, PAK

**Keywords:** hypomagnesemia, hypertension, hba1c, retinopathy, neuropathy

## Abstract

Objective: The study aimed to study the association of hypomagnesemia with diabetic complications in type 2 diabetics.

Materials and method: This cross-sectional study, conducted at a Ghurki Trust Teaching Hospital, spanned from January to June 2023 and included 100 randomly selected diabetic patients aged 30-70. With institutional board approval and informed consent, the study focused on assessing hypomagnesemia, using a standard level of below 1.6 mg/dL, ensuring participant confidentiality and privacy. Data collected through physical assessments were analyzed using IBM SPSS Statistics for Windows, Version 24.0 (Released 2016; IBM Corp., Armonk, New York, United States), including descriptive statistics, analysis of variance (ANOVA), and paired t-test.

Results: A total of 100 diabetic admitted patients were randomly selected for the study ages from 30 to 70 years irrespective of their gender. The mean age of the participants was 53.86±9.74 years. The mean HbA1c of the participants was 8.7±2.32. Forty-eight percent of them had HbA1c less than 8, while 52% had greater than 8 HbA1c. The mean HbA1c in the hypomagnesemia group was 10.8±1.98, while in the normomagnesemia group, it was 8.9±2.2. There were 58.97% of foot ulcers in Group 1, while in Group 2, there were 31.14%. Around 38.46% and 14.75% had neuropathy in Groups 1 and 2, respectively. Nephropathy in Group 1 was 28.20%, while in Group 2, it was 11.47%. Around 69.23% of Group 1 had retinopathy and 37.70% had retinopathy in Group 2. Hypertension was 23.07% in Group 1 and 37.70% in Group 2; moreover, 7.69% and 8.19% had coronary diseases in Groups 1 and 2 accordingly.

Conclusion: The current study concluded that hypomagnesemia was found to have an association with diabetic complications like neuropathy, nephropathy, foot ulcers, and poor glycemic control as evidenced by HbA1c.

## Introduction

During the last several years, diabetes has become increasingly common all over the world. In 2017, researchers calculated a prevalence of 8.4%; by 2022, they anticipated a 9.9% rate, with approximately 629 million cases predicted by the year 2045 [1].

Magnesium is considered an essential electrolyte for any living organism and is the fourth most abundant mineral in the human body [2]. It serves as a cofactor for over 600 vital enzymatic reactions in the human body and an activator for an additional 200 [3]. The decrease in the level of magnesium may result in defective tyrosine kinase activity, causing detrimental effects on the insulin receptors resulting in an impairment of insulin action [4].

Magnesium controls the production of insulin by modulating several channels within beta cells. Adequate cellular Mg2^+^ levels are mandatory at the receptors of insulin for a process called phosphorylation. Magnesium deficiency acts as a direct and major contributor to the development of resistance to insulin. This can be related to the reality that hypomagnesemia has been linked repeatedly to endocrine illnesses, especially type 2 diabetes mellitus (T2DM) [5].

Hypomagnesemia could be linked to the onset or progression of T2DM, although it is increasingly considered that diabetes itself induces hypomagnesemia [6]. Plasma magnesium ion concentrations among T2DM patients are consistently observed to be considerably less than those in non-diabetic healthy individuals [7,8]. Worldwide, between 14% and 48% of people with diabetes also have hypomagnesemia [9-11].

Hypomagnesemia has been linked to a hastened progression of diabetes and a higher likelihood of serious complications in persons with diabetes. Nephropathy, retinopathy, as well as neuropathy are examples of microvascular problems, while cardiac and peripheral arterial disease are examples of macrovascular complications [12-14].

Therefore, given the alarming rise in diabetes prevalence and the tight relationship that exists between metabolic regulation of the disease and disturbed magnesium equilibrium, investigating a possible link between the two is crucial. Identifying the prevalence and contributing factors of hypomagnesemia may help governments and medical experts implement strategies to lessen the issue as well as give further justification for routine hypomagnesemia monitoring of levels of magnesium in all people with T2DM.

In addition, the results may encourage diabetic patients to alter their diets and consume additional magnesium. More timely oral magnesium dietary supplements could reduce or avoid complications associated with diabetes. Therefore, in view of the above studies and limited research investigation in the area, the aim of the current study was to find the association of hypomagnesemia with diabetic complications in type 2 diabetics.

## Materials and methods

Study setting and duration

The present cross-sectional study was conducted in the Department of Medicine at a Ghurki Trust Teaching Hospital from January 2023 to June 2023, spanning a six-month duration, following approval from the institutional review board.

Study participants

A total of 100 diabetic patients admitted to the hospital were randomly selected for the study, with ages ranging from 30 to 70 years. Informed consent was obtained from each participant before their inclusion in the study.

Selection criteria

Participants were included irrespective of gender and were required to have T2DM and fall within the specified age range. Patients with chronic kidney disease, those with autoimmune diseases, those undergoing cancer chemotherapy, individuals below 30 years of age, and those with known cardiovascular diseases were excluded from the study.

Identification of hypomagnesemia

Magnesium levels below 1.6 mg/dL were established as the standard for diagnosing hypomagnesemia. Patients with hypomagnesemia were categorized as cases, while healthy individuals served as controls for comparative analysis.

Data collection and analysis

Data from each participant, including magnesium levels, were collected during the study period. The collected data were subsequently subjected to statistical analysis using IBM SPSS Statistics for Windows, Version 24.0 (Released 2016; IBM Corp., Armonk, New York, United States). Descriptive statistics, including mean values, frequencies, and percentages, were employed to summarize and present the characteristics of the study participants. Mean serum magnesium levels in patients taking different anti-diabetic medications were analyzed by analysis of variance (ANOVA). The paired t-test was employed to evaluate the impact of hypomagnesemia on various complications in both groups. 

Ethical statement

Ethical approval was obtained from the Institutional Review Board (IRB) of Lahore Medical and Dental College (approval number: 13015-56/2022). All participants were assured that their confidentiality and privacy would be maintained throughout the study.

## Results

The study included a cohort of 100 participants, with 44% falling within the age range of 30-50 years, constituting 44 individuals, and 56% in the 51-70-year category, totaling 56 participants. Gender distribution showed 47% were males (n=47), while 53% were females (n=53). The mean age of the participants was 53.86 years (SD=9.74). In terms of the duration of diabetes mellitus (DM), 43% had a duration of less than five years, accounting for 43 individuals, while 57% had a duration of five years or more, totaling 57 participants. The mean HbA1c level was 8.7 (SD=2.32). Among the participants, 48% had HbA1c levels below 8 (n=48), and 52% had levels equal to or above 8 (n=52). These detailed numerical representations provide a comprehensive overview of both numbers and percentages, enhancing the clarity of the demographic and clinical characteristics of the study population (Table 1).

**Table 1:** Demographic characteristic SD: standard deviation; DM: diabetes mellitus

Age (years)	Number	Percentage
30-50	44	44%
51-70	56	56%
Gender		
Male	47	47%
Female	53	53%
Age (mean±SD)	53.86±9.74	
Duration of DM (years)		
<5	43	43%
≥5	57	57%
HbA1c (mean±SD)	8.7±2.32	
<8	48	48%
≥8	52	52%

Table 2 shows the mean serum magnesium level for patients taking different diabetic medications. SGLT2 inhibitors have the lowest mean level (1.9 mg/dL) with 21 patients, followed by metformin (2.2 mg/dL) with 26 patients. Insulin (1.8 mg/dL) and gliptin (1.7 mg/dL) have lower mean levels than metformin, but data for statistical comparison (p-value) is only shown for SGLT2 inhibitors.

**Table 2:** Comparison of mean serum magnesium levels in patients taking different anti-diabetic medications

Anti-diabetic medication	Number of patients	Mean serum magnesium level (mg/dL)	Distribution probability (%)	P-value
SGLT2 inhibitor	21	1.9	21	0.023
Metformin	26	2.2	26	
Insulin	35	1.8	35	
Gliptin	18	1.7	18	

Table 3 highlights the complications associated with hypomagnesemia. The mean HbA1c in the hypomagnesemia group was 10.8±1.98, while in the normomagnesemia group, it was 8.9±2.2. There were 58.97% of foot ulcers in Group 1, while in Group 2, there were 31.14%. Around 38.46% and 14.75% had neuropathy in Groups 1 and 2, respectively. Nephropathy in Group 1 was 28.20%, while in Group 2, it was 11.47%. Around 69.23% of Group 1 had retinopathy and 37.70% had retinopathy in Group 2. Hypertension was 23.07% in Group 1 and 37.70% in Group 2; moreover, 7.69% and 8.19% had coronary diseases in Groups 1 and 2 accordingly. The mean HbA1c level was 10.8, with associated complications such as 23 cases of foot ulcers, 15 cases of neuropathy, 11 cases of nephropathy, 27 cases of retinopathy, five cases of proteinuria, three cases of coronary artery disease, and nine cases of hypertension. The calculated paired t-test statistics revealed a t-value of 0.326 and a corresponding p-value of 0.754. The nonsignificant p-value suggests that there is no statistically significant difference in the complications observed in individuals with hypomagnesemia in Group 1, providing insights into the relationship between magnesium levels and diabetic complications within the specified parameters.

**Table 3:** Complications of hypomagnesemia

Complications	Group 1 hypomagnesemia (N=39)	Group 2 hypomagnesemia (N=61)
HbA1c (mean)	10.8	8.9
Foot ulcers	23	19
Neuropathy	15	9
Nephropathy	11	7
Retinopathy	27	23
Proteinuria	5	3
Coronary artery disease	3	5
Hypertension	9	23
Paired t-test		
t	0.326	
p	0.754	

## Discussion

The relationship between serum magnesium levels and diabetes has been extensively studied, revealing a significant association between magnesium deficiency and various endocrine and metabolic disorders, with DM being the most common [15]. Hypomagnesemia in individuals with T2DM can result from multiple factors, including inadequate intake, diabetes-related complications such as gastroparesis and autonomic dysfunction-related diarrhea, increased glomerular hyperfiltration, renal magnesium loss, metabolic acidosis, glycosuria due to osmotic diuresis, and impaired magnesium resorption in the kidneys due to diabetes-related insulin resistance [16,17].

Among the 100 patients included in the study, 44% were aged between 30 and 50 years, while 56% were aged between 51 and 70 years. The gender distribution was 47% males and 53% females. The mean age of the participants was 53.86±9.74 years, with 43% having diabetes for less than five years and 57% for more than five years. The mean HbA1c was 8.7±2.32, with 48% having HbA1c less than 8 and 52% greater than 8. Comparing these findings with a similar study by Hamarshih et al., which reported an average age of 56.2±10.8 years and associations with factors like smoking, overweight status, and hypertension, provides a broader context [2].

The study identified that one in 10 participants had low magnesium levels, with females, individuals with an HbA1c of 8%, and those with a prior diagnosis of diabetic retinal degeneration showing increased susceptibility at higher risk [16]. Notably, a substantial relationship between hypomagnesemia and HbA1c levels (p-value=0.019) was observed. Individuals with HbA1c of ≥8% had a hypomagnesemia prevalence of 14.9%, compared to 7.2% in those with HbA1c lower than 8%, emphasizing the adverse link between magnesium levels and sugar regulation [18].

Another study by Dasgupta et al. reported that individuals with hypomagnesemia had a higher HbA1c average of 11.9%, compared to the control group's average of 9.8%. Those with hypomagnesemia showed a higher prevalence of complications such as retinopathy, microalbuminuria, macroalbuminuria, foot ulcers, and neuropathy. Intriguingly, hypomagnesemia was associated with a lower prevalence of cardiovascular disease (17.6% vs. 39%) overall and in the subgroup aged 50 and above (27% vs. 25%) [15]. These findings contribute valuable insights into the intricate relationship between magnesium levels and diabetic complications, emphasizing the need for further exploration and understanding of this association.

Limitations

The sample size of 100 diabetic patients from a single teaching hospital may not fully represent the diverse diabetic population. The reliance on a specific age range (30-70 years) further limits the generalizability of our findings. The absence of a control group without diabetes hinders the comparison of magnesium levels in diabetic and non-diabetic individuals. Despite these limitations, our study provides a foundation for further exploration into the intricate relationship between magnesium status and diabetic complications.

Future perspectives

Future research should involve exploring interventions to address hypomagnesemia in diabetic patients, aiming to mitigate the associated complications. Investigating the potential benefits of magnesium supplementation or dietary modifications may provide insights into preventive measures or therapeutic strategies. Longitudinal studies could further elucidate the causal relationships between hypomagnesemia and specific diabetic complications, contributing to the development of targeted and effective interventions in the management of diabetes-related health issues.

## Conclusions

The recent investigation involved a cohort of 100 randomly selected diabetic inpatients aged 30-70 years, revealing notable associations between hypomagnesemia and various diabetic complications. The participants, with a mean age, demonstrated a range of glycemic control. The hypomagnesemia group exhibited a higher mean HbA1c compared to the normomagnesemia group. Significant differences were noted in the prevalence of complications in the hypomagnesemia group compared to the normomagnesemia group. The study underscores the association between hypomagnesemia and diabetic complications, emphasizing its impact on neuropathy, nephropathy, foot ulcers, and glycemic control.

## References

[1] Cho NH, Shaw JE, Karuranga S, Huang Y, da Rocha Fernandes JD, Ohlrogge AW, Malanda B (2018). IDF Diabetes Atlas: global estimates of diabetes prevalence for 2017 and projections for 2045. Diabetes Res Clin Pract.

[2] Nasri H (2014). Consequences of hypomagnesemia in type 2 diabetes mellitus patients. J Renal Inj Prev.

[3] de Baaij JH, Hoenderop JG, Bindels RJ (2015). Magnesium in man: implications for health and disease. Physiol Rev.

[4] Mahalle N, Garg MK, Kulkarni MV, Naik SS (2014). Relation of magnesium with insulin resistance and inflammatory markers in subjects with known coronary artery disease. J Cardiovasc Dis Res.

[5] Ramadass S, Basu S, Srinivasan AR (2015). SERUM magnesium levels as an indicator of status of diabetes mellitus type 2. Diabetes Metab Syndr.

[6] Feng J, Wang H, Jing Z, Wang Y, Cheng Y, Wang W, Sun W (2020). Role of magnesium in type 2 diabetes mellitus. Biol Trace Elem Res.

[7] Kim DJ, Xun P, Liu K, Loria C, Yokota K, Jacobs DR Jr, He K (2010). Magnesium intake in relation to systemic inflammation, insulin resistance, and the incidence of diabetes. Diabetes Care.

[8] Pham PC, Pham PM, Pham SV, Miller JM, Pham PT (2007). Hypomagnesemia in patients with type 2 diabetes. Clin J Am Soc Nephrol.

[9] Gommers LM, Hoenderop JG, Bindels RJ, de Baaij JH (2016). Hypomagnesemia in type 2 diabetes: a vicious circle?. Diabetes.

[10] Pokharel DR, Khadka D, Sigdel M, Yadav NK, Kafle R, Sapkota RM, Jha SK (2017). Association of serum magnesium level with poor glycemic control and renal functions in Nepalese patients with type 2 diabetes mellitus. Diabetes Metab Syndr.

[11] Waanders F, Dullaart RP, Vos MJ, Hendriks SH, van Goor H, Bilo HJ, van Dijk PR (2020). Hypomagnesaemia and its determinants in a contemporary primary care cohort of persons with type 2 diabetes. Endocrine.

[12] Lu J, Gu Y, Guo M, Chen P, Wang H, Yu X (2016). Serum magnesium concentration is inversely associated with albuminuria and retinopathy among patients with diabetes. J Diabetes Res.

[13] Arpaci D, Tocoglu AG, Ergenc H, Korkmaz S, Ucar A, Tamer A (2015). Associations of serum magnesium levels with diabetes mellitus and diabetic complications. Hippokratia.

[14] Zhang Y, Li Q, Xin Y, Lv W, Ge C (2018). Association between serum magnesium and common complications of diabetes mellitus. Technol Health Care.

[15] Dasgupta A, Sarma D, Saikia UK (2012). Hypomagnesemia in type 2 diabetes mellitus. Indian J Endocrinol Metab.

[16] Hamarshih M, Hamshari S, Nazzal Z, Snobar F, Mletat R, Abu-Mazen O, Maraqa B (2022). Hypomagnesemia and poor glycemic control among type 2 diabetic patients: a cross-sectional study. Indian J Endocrinol Metab.

[17] Joy SS, George TP, Siddiqui K (2019). Low magnesium level as an indicator of poor glycemic control in type 2 diabetic patients with complications. Diabetes Metab Syndr.

[18] Hyassat D, Al Sitri E, Batieha A, El-Khateeb M, Ajlouni K (2014). Prevalence of hypomagnesaemia among obese type 2 diabetic patients attending the National Center for Diabetes, Endocrinology and Genetics (NCDEG). Int J Endocrinol Metab.

